# An Informative Discussion for School Nurses on COVID-19 mRNA Vaccine

**DOI:** 10.1177/1942602X21999606

**Published:** 2021-04-07

**Authors:** Jenny Gordon, Mark Reynolds, Elizabeth Barnby

**Affiliations:** Pediatric Registered Nurse, Huntsville, AL; Clinical Associate Professor, College of Nursing, University of Alabama in Huntsville, Huntsville, AL; Clinical Associate Professor, College of Nursing, University of Alabama in Huntsville, Huntsville, AL

**Keywords:** COVID-19, mRNA vaccine, advocacy, education

## Abstract

School nurses are advocates, caregivers, and teachers. It is the responsibility of school nurses to understand current prevention and treatment options. In understanding how and why coronavirus disease 2019 (COVID-19) mRNA vaccines work, school nurses are in a trusted position to explain and advocate vaccination to students and their caregivers. The messenger ribonucleic acid (mRNA) vaccine is a product of the latest scientific and medical technology. A better understanding of how and why this vaccination is effective may prevent vaccination hesitancy and provide reassurance to those choosing to accept vaccination. In December 2020, the National Association of School Nurses publicized its support for vaccination against COVID-19. As the COVID-19 pandemic lingers school nurses will step toward the front line to aid in the abatement of poor public health outcomes that may be severely affecting their schools, students, and caregivers.

## Introduction

Nurses have been recognized as the most trusted profession for the 18th consecutive year as evaluated by the Gallup Poll (Reinhart, 2020). School nurses are advocates, caregivers, and teachers. When students and caregivers are at their most vulnerable, school nurses are there to help them make decisions and provide essential nursing services. Immunization through vaccination is one of the greatest medical and scientific achievements of the 20th century (National Foundation for Infectious Disease, 2020). It has saved countless lives, prevented chronic disability, and provided protection against deadly illnesses. As science and medicine have advanced, so have techniques and processes of vaccination. Viruses and bacteria are constantly changing. Science and medicine must evolve alongside them to provide effective countermeasures. The coronavirus disease 2019 (COVID-19) global pandemic is a war against a new, potentially deadly, and debilitating pathogen. Out of the global pandemic a new 21st century type of vaccination has arrived. In understanding how and why COVID-19 messenger ribonucleic acid (mRNA) vaccines work, school nurses are in a trusted position to explain and advocate vaccination to students and their caregivers.

## mRNA Scientific and Medical Technology

The mRNA vaccine is a product of the latest scientific and medical technology (Pardi et al., 2018). This vaccine was made possible by the shared resources and contributions of scientists worldwide. The mRNA COVID-19 vaccine is elegant in the simplicity of its concept. Due to its novel approach, there exists a lack of comprehension and familiarity with its process. A better understanding of how and why this vaccination is effective may prevent vaccination hesitancy and provide reassurance to those choosing to accept vaccination.

**Figure 1.**
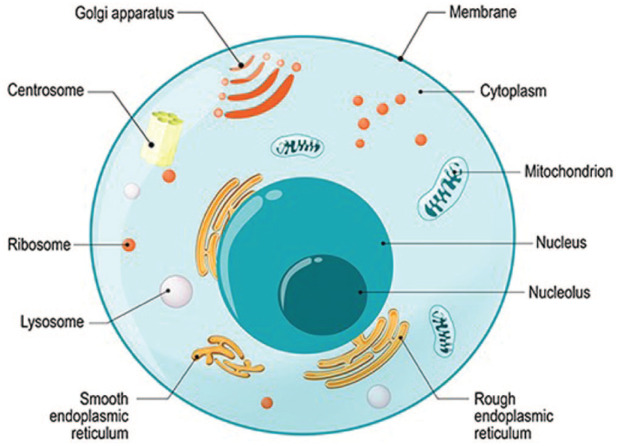
Cross Section of Human Cell *Source.* iStock.com/ttsz

A brief review of basic cellular biology provides foundational knowledge of the role mRNA plays within the human body. The human body is composed of billions of individual cells. Each cell has a nucleus at the center, ribosomes both free floating and attached to the mazelike endoplasmic reticulum, and a plasma membrane. The plasma membrane serves as a barrier and access point for entry and exit within the cell (O’Connor et al., 2011). Various other structures including lysosomes, mitochondria, centrioles, the Golgi apparatus, and cytoplasm are also found within each cell. At the center of each cell, the nucleus contains deoxyribonucleic acid (DNA; see Figure 1). DNA is the template for RNA. This is to say that DNA holds and provides the recipes that become different types of RNA, including mRNA. It is important to note that this process does not occur in reverse. mRNA cannot become DNA and mRNA does not make changes to DNA. After the mRNA is produced, it travels out to the ribosomes. Ribosomes are akin to production factories for proteins. The ribosome factory “reads” the mRNA directions and synthesizes specific types of proteins. Once produced, these proteins go on to carry out their specific cellular and biologic functions (Komaroff, 2020).

Viruses are the ultimate saboteur. They are varied in their physical appearance and share a common goal. This goal is to use humanity’s own cellular machinery to aid their reproduction and spread. COVID-19 is no different. The virus sneaks through the immune defenses to penetrate inside human cells. COVID-19 is a specific type of virus known as a beta RNA virus. Beta RNA viruses cause more serious symptoms and sequela when compared with their alpha RNA virus counterparts (Velavan & Meyer, 2020). As an RNA virus, once COVID-19 gains entry into the interior of the cell, it covertly delivers its own mRNA codes to the ribosomes. The ribosome factory reads the COVID-19 mRNA. From these forged instructions, the ribosomes produce proteins that the virus needs to proliferate and infect the human body. People who have contracted COVID-19 have had their own cellular machinery deceived by COVID-19 viral mRNA. The integrity of the cell is breached by COVID-19. As previously explained, vaccine mRNA does not breach the nucleus and does not cause changes to DNA (Centers for Disease Control and Prevention [CDC], 2020).

When COVID-19 became a pandemic, scientists worldwide rushed to amass knowledge about every aspect of the virus. This included mapping the COVID-19 genetic sequence. This genome sequence was shared publicly. Knowledge sharing between researchers facilitated the identification of COVID-19 structural proteins. Recognition of the segments of viral RNA that correlate with specific proteins is key in producing an mRNA vaccine. There are four types of different proteins expressed on the surface of COVID-19 (Luk et al., 2019). The most “well-known” of these proteins is the spike protein, labeled by researchers as the (S) protein. This protein produces the unique characteristic of coronaviruses that imparts the notorious crown-like shape. It is ultimately this protein code that has been targeted for laboratory replication. The sequence of mRNA that is responsible for the production of the spike protein is the biologically produced code or mRNA delivered in the vaccine. Just as actual viral mRNA can feed a substituted set of instructions to the ribosome, the vaccine also utilizes this stealthy technique (Walsh et al., 2020). Researchers and scientist have taken heed of viral subterfuge and copied one of its effective methods.

## COVID-19 Immunization Through Vaccination

A vaccine is a mixture that contains antigens originating from a specific microorganism of an infectious disease to provoke an immune response (F.A. Davis, n.d.). The elicited immune response allows the body to produce special proteins, known as antibodies, which will fight against future potential infections from the same microorganism. The National Association of School Nurses (NASN, 2020) supports immunization through vaccination to reduce the incidence of vaccine preventable diseases. A vaccine is made to stimulate the body to respond to it by producing an antibody. Inflammation is the body’s response to foreign proteins. COVID-19 mRNA vaccine side effects are inflammatory in nature. When the immune system encounters foreign proteins (the components that make up viruses and germs) it responds with an accumulation of immune cells. The body sends its defense cells to fight the foreign invader. The immune response is a cascade of events that release inflammatory mediators. These inflammatory immune mediators produce local symptoms such as injection site pain and swelling or systemic symptoms including fever and fatigue (Hervé et al., 2019). People vary in the responsiveness of their immune system to vaccines and their components. The severity of the body’s physical reaction to a vaccine is termed its *reactogenicity*. It is important that a vaccine produce signs of reactogenicity as this translates to the vaccine eliciting a protective immune response (Hervé et al., 2019). Different types of vaccines, and the components they are made of, stimulate different levels of reactogenicity or side effects.

At this time, the known side effects of mRNA vaccination include “injection site pain, fatigue, headache, muscle pain, chills, joint pain, fever, injection site swelling, injection site redness, nausea, malaise, and lymphadenopathy” (U.S. Food and Drug Administration, 2020, p. 7). The most common side effects reported in clinical trials were subjectively described by recipients as mild to moderate pain in the muscle receiving the vaccination, fatigue, and headache (Polack et al., 2020; see Table 1). The Moderna mRNA clinical trial reported that the majority of side effects resolved by Day 2 postvaccination (Baden et al., 2020). By and large, the vaccine is well tolerated.

**Table 1.** Moderna and Pfizer Clinical Trial Vaccine Most Common Side Effects

Side Effect	Moderna Clinical Trial (%)	Pfizer Clinical Trial (%)
Pain at injection site	91.6	84.1
Fatigue	68.5	62.9
Headache	63.0	55.1
Muscle pain	59.6	38.3
Joint pain	44.8	23.6
Chills	43.4	31.9

*Note.* Food and Drug Administration Briefing Document: Moderna COVID-19 Vaccine, 2020; Food and Drug Administration Briefing Document: Pfizer-BioNTech COVID-19 Vaccine, 2020.

It is true that some individuals who contract COVID-19 are asymptomatic or have only mild illness. It is also true that COVID-19 can lead to permanent organ damage or even death (CDC, 2021). Possible effects of COVID-19 infection include fever, cough, vomiting, diarrhea, temporary loss of taste and smell, pneumonia, pulmonary fibrosis, acute respiratory distress syndrome, pulmonary emboli, chest pain, cardiomyopathy, multisystem inflammatory syndrome, body aches, fatigue, multiple organ failure, hypercoagulability, and death (Polack et al., 2020). At the current time, there is no system or test in place to identify who will have a mild, moderate, or severe course of the disease. The CDC provides up-to-date state and national statistics about positive cases reported through public health, commercial, and clinical laboratories; mild/moderate illness reported through outpatient and emergency department visits; and severe cases leading to hospitalization and deaths through the CDC’s (2021) COVID Data Tracker.

Vaccine hesitancy is considered the concern to decide on vaccinating self or children (Salmon et al., 2015). In the United States, specifically among minorities and those of low socioeconomic status, vaccine hesitance is common due to various risks perception. Risk perceptions such as the powerlessness to regulate adverse events following immunization, the manufactured nature of vaccines, the uncertainty of effects, and the obligatory requirement of vaccines result in individuals “perceiving the risk of vaccines to be greater than they actually are” (Salmon et al., p. S392). In making a decision to accept vaccination, it is important to know how well the vaccine works to prevent the target disease. To meet the criteria for emergency use authorization (EUA) by the U.S. Food and Drug Administration, mRNA vaccines had to demonstrate an efficacy rate greater than or equal to 50% during clinical trials (U.S. Food and Drug Administration, 2020). The efficacy rate as reported by clinical trials from 7 to 14 days after the receipt of the second vaccine dose is 95% for the Pfizer-BioNTech vaccine (Polack et al., 2020) and 94.1% for the Moderna vaccine (Baden et al., 2020). This short-term efficacy rate far exceeds the 50% required for EUA standards. The remaining question, and one that will be answered with the ongoing clinical trial analysis, is how long the mRNA efficacy rate of approximately 95% lasts.

## Connections to the Framework

As the COVID-19 pandemic lingers, school nurses will step toward the front line to aid in the abatement of poor public health outcomes as well as interrupted student attendance that may be severely affecting their schools, students, and caregivers. In conjunction with the NASN’s (2016) Framework for 21st Century School Nursing Practice, the school nurse’s role in explaining and advocating for vaccination relates to all of the framework’s principles. The Standards of Practice and Leadership principles include school nurses learning and understanding evidence-based practices for the COVID-19 disease process as well as the COVID-19 mRNA vaccination components. Care Coordination includes school nurses educating students and caregivers about their opportunities for COVID-19 mRNA vaccinations. Community/Public Health and Quality Improvement will remain a focus of school nurses as they are qualified to host vaccination clinics in their schools and may be called on to track data related to the impact of pandemic on school-related measures such as attendance. School nurses have access to a large population of students and caregivers, which provides them the opportunity to educate them and vaccinate them, if applicable.

## Conclusion

COVID-19 has carved a path of destruction across the globe. No country or person has remained unaffected by this pandemic. As of February 11, 2021, more than 2.4 million lives have been lost to COVID-19 (Worldometer, 2021). Economists Cutler and Summers (2020) have estimated the financial cost of COVID-19 at greater than $16 trillion for the United States alone. These statistics only represent the measurable burden of disease. Rates of productivity and emotional and physical stress have similarly suffered. Long-term effects of obstructive pulmonary disease and organ damage are unknown. To date, treatment for COVID-19 remains limited and in short supply. Primary care, through vaccination and prevention of disease, is a mainstay of medicine. mRNA COVID-19 vaccination has the potential to break the pandemic. Vaccination has a rich history of saving lives and preventing disability. The COVID-19 virus has stolen lives, employment, freedom, and emotional stability from people of all ages and ethnicities. The best and brightest of researchers and scientists have collaborated to present humanity with a safe and effective solution to the current crisis.

School nurses must educate themselves on COVID-19, mRNA vaccination, and vaccine hesitancy. It is critical to have a knowledge base to draw from when practicing shared decision-making, addressing student or caregiver concerns, and making recommendations. As nurses are the most trusted profession, maintaining our veracity and encouraging open communication is paramount. A clear risk–benefit analysis along with an honest discussion of potential side effects of immunization through vaccination is essential. Table 2 provides a listing of resources school nurses can utilize to aid in the education and understanding of COVID-19, education and understanding mRNA vaccines, and help reduce vaccine hesitancy. ▪

**Table 2.** Resources for School Nurses

Understanding COVID-19	*John Hopkins University and Medicine:* Understanding the COVID-19 Pandemic https://coronavirus.jhu.edu/covid-19-basics/understanding-covid-19
	*KidsHealth:* Understanding Coronavirus (COVID-19) https://kidshealth.org/en/parents/coronavirus-landing-page.html?WT.ac=p-ra
	*Centers for Disease Control and Prevention:* COVID Data Tracker https://covid.cdc.gov/covid-data-tracker/#datatracker-home
	*Cedars Sinai:* Understanding COVID-19 Vocabulary https://www.cedars-sinai.org/blog/covid-19-vocabulary.html
	*Centers for Disease Control and Prevention:* COVID-19 Frequently Asked Questions https://www.cdc.gov/coronavirus/2019-ncov/faq.html
Understanding COVID mRNA vaccines	*COVID-19 Vaccines:* Get the Facts, Mayo Clinic https://www.mayoclinic.org/diseases-conditions/coronavirus/in-depth/coronavirus-vaccine/art-20484859
	*Food and Drug Administration Briefing Document:* Pfizer-BioNTech COVID-19 Vaccine https://www.fda.gov/media/144245/download
	*Food and Drug Administration Briefing Document:* Moderna COVID-19 Vaccine https://www.fda.gov/media/144434/download
	*Yale School of Medicine:* Understanding COVID-19: How Vaccines Work With Your Immune System https://medicine.yale.edu/media-player/5993/
	*Centers for Disease Control and Prevention:* Understanding How the COVIDE-19 Vaccines Work https://www.cdc.gov/coronavirus/2019-ncov/vaccines/different-vaccines/how-they-work.html
Reducing Vaccine Hesitancy	*National Institutes of Health:* COVID-19 Vaccination Communication: Applying Behavioral and Social Science to Address Vaccine Hesitancy and Foster Vaccine Confidence https://obssr.od.nih.gov/wp-content/uploads/2020/12/COVIDReport_Final.pdf
	*Centers for Disease Control and Prevention:* Building Confidence in COVID-19 Vaccines Among Your Patients https://www.cdc.gov/vaccines/covid-19/downloads/VaccinateWConfidence-TipsForHCTeams_508.pdf
	*World Health Organization:* Strategies for Addressing Vaccine Hesitancy—A Systematic Review https://www.who.int/immunization/sage/meetings/2014/october/3_SAGE_WG_Strategies_addressing_vaccine_hesitancy_2014.pdf
	*American Pharmacists Association:* Vaccine Hesitancy: Understanding and Addressing Vaccine Hesitancy During COVID-19 https://www.pharmacist.com/sites/default/files/audience/APhACOVID-19VaccineHesitancy_1120_web.pdf
